# Leiomyosarcoma of Inferior Vena Cava and Right Atrium with Ascites and Jaundice: A Case Report

**Published:** 2016-10-01

**Authors:** Roshanak Hasheminasab Zavareh, Hassan Riahi Beni, Aida Iranpour, Mojgan Alam Samimi, Alireza Sadeghipour, Seyedeh Nina Alavi Niakou

**Affiliations:** 1 Department of Internal Medicine, Rasoul Akram General Hospital, Iran University of Medical Sciences, Tehran, Iran; 2 Department of Cardiovascular Diseases, Rasoul Akram General Hospital, Iran University of Medical Sciences, Tehran, Iran; 3 Department of Hematology and Oncology, Rasoul Akram General Hospital, Iran University of Medical Sciences, Tehran, Iran; 4 Department of Pathology, Rasoul Akram General Hospital, Iran University of Medical Sciences, Tehran, Iran

**Keywords:** Leiomyosarcoma, Cardiac mass, Ascites, Inferior vena cava

## Abstract

Leiomyosarcoma is one of the soft tissue sarcomas that could originate from different parts of body and are mostly presented as retropritoneal mass. Leiomyosarcomas of vascular origin are particularly rare tumors occurring mainly in inferior vena cava (IVC). Here, we report the case of a 35-year-old male patient who presented with ascites and jaundice. Further evaluation revealed large hepatic and cardiac masses with extension to IVC. Since it was not possible to determine the point of origin of leiomyosarcoma, the patient received chemotherapy under diagnosis of metastatic leiomyosarcoma but unfortunately passed away.

## Introduction

 Leiomyosarcomas, which are characterized by smooth muscle differentiation, can be found throughout the body including the retroperitoneum and any location where there is a vein.^1^ The presentation of the tumor is different, depending on the site and origin of the tumor. Mostly presents as a large retropritoneal mass and the involvement of IVC but cardiac chambers are extremely rare. Here, we report a case presented with inferior vena cava (IVC) mass associated with hepatic and right atrium tumors.

## CASE PRESENTATION

 A 35-year-old male patient presented with abdominal and lower extremity swellings. He stated that abdominal swelling started 15 days ago and then he observed an increase in the swelling. Meanwhile, six days before admission, he noticed swelling in both legs. He also complained of jaundice, dyspnea on exertion, loss of appetite and malaise. Abdominal ultrasound was performed and revealed severe ascites, splenomegaly and a hypoecho mass in the right liver lobe with extension to midline. On physical examination, the patient was conscious and a generalized icterus was seen. Abdominal distention, bilateral decreased breath sounds in basilar lung regions and pitting edema of both lower extremities were detected but the remainder of examination was normal.

**Figure 1:**
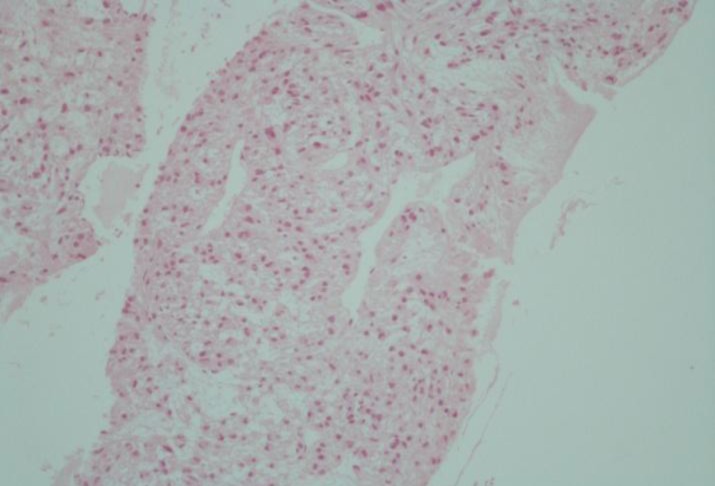
Pleomorphic, plump, spindle and polyhedral cells arranged as short bundles and fascicles with capillary-sized vascular channels between them .The tumor cells show nuclear-to- cytoplasmic ratio with moderate nuclear pleomorphism and hyperchromasia. Cytoplasmic borders are indistinct (Hematoxylin and Eosin, X100).

**Figure 2:**
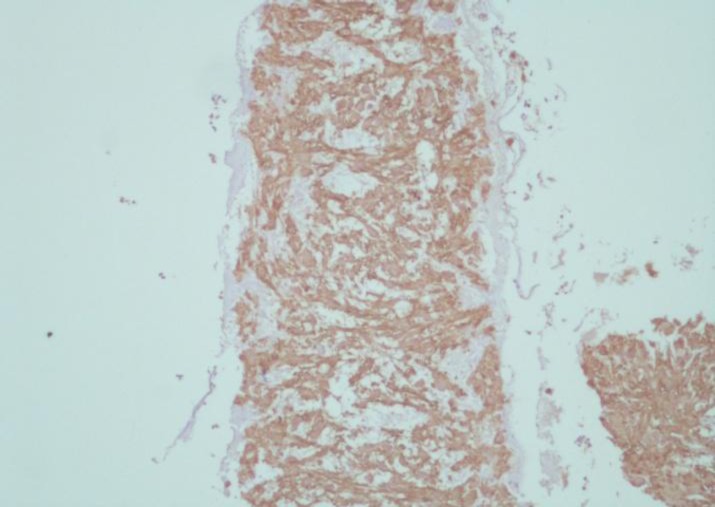
Tumor cells show diffuse cytoplasmic reactivity with Anti-Actin (muscle) (Clone: HHF35, monoclonal Mouse Anti-Human, X100).

On evaluation, ascitic fluid analysis revealed the high serum*-*ascites albumin gradient (SAAG) fluid and triphasic abdominal computed tomography (CT) confirmed a large mass in the right liver lobe with changes in inferior vena cava (IVC) and hepatic veins suggestive of tumor extension or thrombosis (Figures 1, 2). Doppler ultrasound of hepatic and portal veins showed no thrombosis. 

For further evaluation of generalized edema and cardiac function, transthoracic echocardiography was done and showed a large mass 40*40 millimeters in the right atrium and small pericardial effusion. Laboratory tests were remarkable for abnormal liver function test in hepatocellular pattern and prolonged prothrombin time (PT) in association with hypochrome microcytic anemia (Table 1).

**Figure 3:**
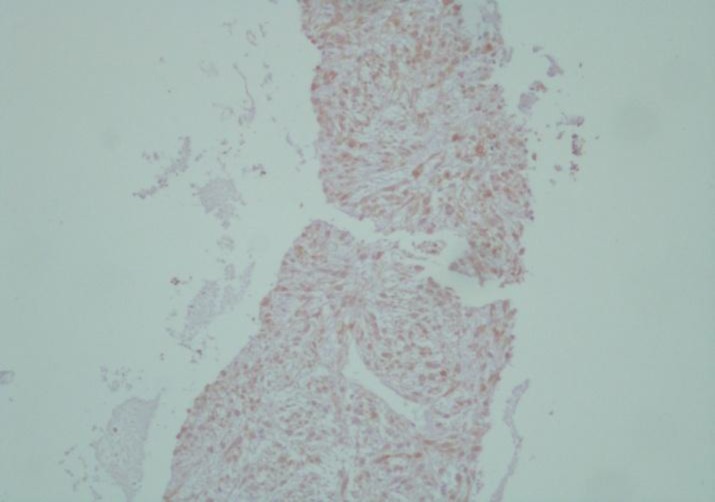
High proliferation capacity of tumor cells demonstrated by staining with anti-Ki67 Antigen (Clone: MIB-1 monoclonal mouse anti-human, X100).

**Figure 4:**
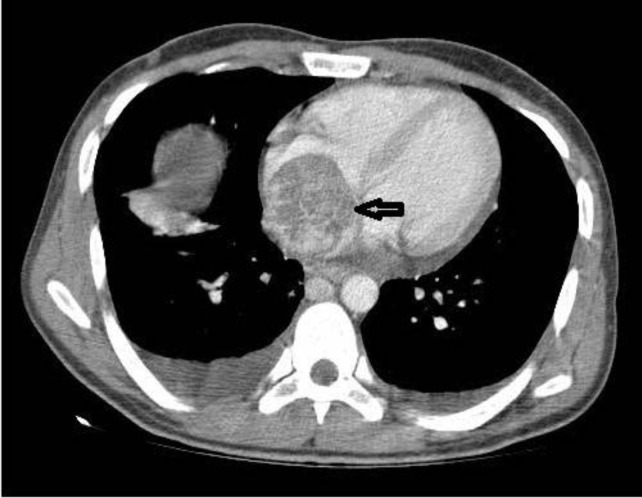
Contrast-enhanced chest computed tomography revealed large heterogeneous mass in right atrium (black arrow) and bilateral pleural effusion.

**Figure 5:**
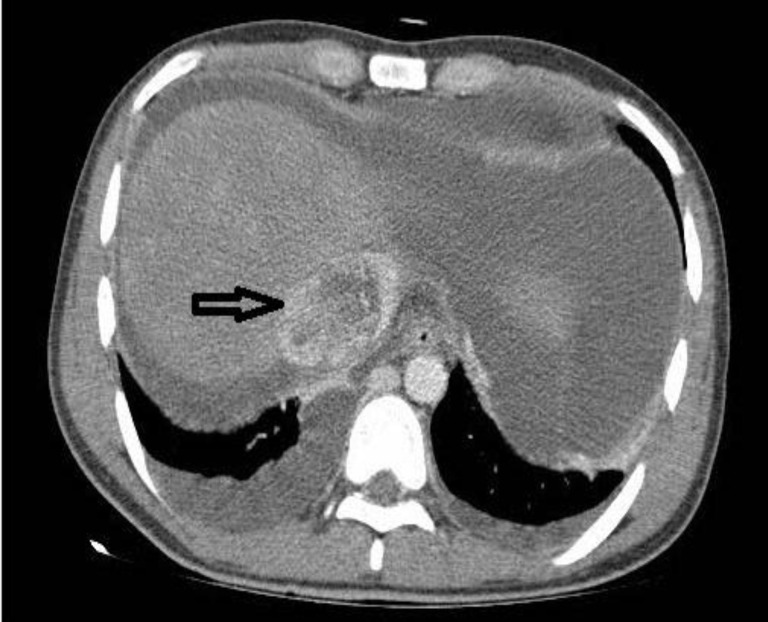
Contrast-enhanced abdominal computed tomography showed extension of the mass to IVC (black arrow) and ascitis.

Femoral vein catheterization was done to try biopsy of IVC mass, femoral vein catheterization was done to perform IVC mass biopsy but tortuous course of IVC made it impossible. Due to the location of hepatic mass, transjugular or percutaneous liver biopsy was not possible. So, CT-guided biopsy from IVC mass was performed. Pathologic examination and immunohistochemistry study revealed high grade leiomyosarcoma (Figure 3, 4, 5).

The patient received chemotherapy regimen including gemcitabine and docetaxel under diagnosis of metastatic leiomyosarcoma. Due to tumor extension, patient was not a suitable candidate for surgery or radiotherapy; so, chemotherapy continued. He then developed hepatic encephalopathy and liver failure during hospitalization and unfortunately passed away. First degree relative of patient had given a written consent for publishing information**.**

## Discussion

 Sarcomas are rare and heterogeneous group of malignant tumors of mesenchymal origin that comprise approximately 1 % of all adult malignancies. Approximately, 80 % of sarcomas originate from soft tissue and the rest from bone.^1^ The WHO classifies most soft tissue sarcomas according to presumptive tissue of origin that one of them is leiomyosarcoma.^2^ Leiomyosarcomas, which are characterized by smooth muscle differentiation, can be found throughout the body including the retroperitoneum, any location where there is a vein and the uterus. The most common presentation is an asymptomatic mass. Mechanical symptoms referable to pressure, traction, or entrapment of nerves or muscles may also be present.

This case demonstrates the difficulty in distinguishing the origin of the tumor and its uncommon presentations. As we mentioned above, our patient presented with ascites and jaundice and further evaluation revealed IVC mass associated with hepatic and cardiac tumors. Leiomyosarcoma of the inferior vena cava (IVC) is the most common primary malignancy of the IVC and has an extremely poor prognosis.^3^ The most common presenting symptom is a pain in the right flank and other presentations are edema of the lower extremities, Budd-Chiari syndrome with hepatomegaly, ascites, and jaundice.^4^^,^^5^ Systemic metastasis occurs in less than 50 percent of patients in late stages and most common sites are liver, lung, lymph nodes or bone^6^ and propagation of the tumor to right atrium occurs in less than 20 percent of patients.^7^

Metastatic soft tissue sarcomas are largely incurable but up to 20% of patients who achieve a complete response become long-term survivors. The therapeutic intent, therefore, is to produce a complete remission with chemotherapy (<10%) and/or surgery (30–40%) although the efficacy of chemotherapy and radiotherapy is extremely low in leiomyosarcoma.^8^ Surgical resection of metastases, whenever possible, is an integral part of the management. The two most active chemotherapeutic agents are doxorubicin and ifosfamide. Gemcitabine with or without docetaxel has become an established second-line regimen and is particularly active in patients with leiomyosarcoma.^9^^,^^10^

**Table 1:** Laboratory tests

**Variable**	**On ** **admission**	**Variable**	**On ** **admission**	**Variable**	**On ** **admission**
WBC	5600	ALP	370	INR	2.2
Hb	13.1	Bili Total	6	AFP	1.3
Platelet	154000	Bili Direct	2.1	Β-hCG	<1
Ferritin		Serum Alb	3.1	ESR	50
AST	167	Creatinine	1.2	CRP	62
ALT	182	LDH	471		

## CONCLUSION

 Since leiomyosarcoma of vascular origin is an uncommon finding and metastasis to heart chambers is even more uncommon, we aimed to report this rare presentation of sarcomas of vascular origin.
